# Supplementary material for: Pioglitazone-Primed Mesenchymal Stem Cells Stimulate Cell Proliferation, Collagen Synthesis and Matrix Gene Expression in Tenocytes
Source: Int J Mol Sci. 2019 Jan 22;20(3):472. doi: 10.3390/ijms20030472 (PMC6387004; doi:10.3390/ijms20030472)
Supplement: Supplementary file 1 [file ijms-20-00472-s001.pdf]

Supplementary Methods: Immunoblotting and Animal Experiments.

Cellular proteins were obtained via the fractionation of MSC and Pio-MSC lysates with 10% sodium dodecyl sulfate polyacrylamide gel electrophoresis (SDS-PAGE) under reducing conditions. The protein bands were transferred onto a polyvinylidene fluoride membrane and analyzed using anti-AKT (Cell Signaling, Los Angeles, CA, USA), anti-phospho AKT (Thr308, Cell Signaling), anti-p44/42 mitogen-activation protein kinase (MAPK; Thr202/204; Cell Signaling), polyclonal anti-phospho p44/42 MAPK (Cell Signaling), and anti-beta-actin antibodies (Abcam) at 4 °C overnight. Before probing, non-specific binding was blocked by incubation with 5% bovine serum albumin (BSA) in TBST (10 mM Tris, pH 8.0, 150 mM sodium chloride [NaCl], and 0.5% Tween-20) for 60 min at room temperature. Membranes were washed four times for 10 min each and incubated with horseradish peroxidase-linked goat anti-rabbit secondary antibody (1:3000; Abcam) at room temperature for 1 h. Blots were washed four times with TBST and developed with the enhanced chemiluminescence (ECL) system (Amersham Biosciences, Waltham, MA, USA) in accordance with the manufacturer's protocols and were quantified using ImageJ software (NIH).

Six 10-week-old male Sprague Dawley rats (Orient Bio, Seongnam, Korea) were used in the present study. Rats were divided into MSC group and Pio-MSC group. Each rat was anesthetized with 2% isoflurane. A longitudinal skin incision was made over the achilles tendon with surgical blade. After tendon exposure, 0.6 × 6 mm-sized window defects were made in the central portion of both Achilles tendons using surgical blades assembled on a custom made plastic rack. [S1] The defect of one leg was filled with a 60 μL volume of mixtures with 5×10^5 MSCs or Pio-MSCs and fibrin glue (Greenplast Kit, Green Cross, Seoul, Korea) using dual syringe injection system according to the group. The defect of the opposite side was filled with a same volume of mixtures with phosphate buffered saline and fibrin glue and used as a control for MSC or Pio-MSC injected tendons. Two weeks after MSC and Pio-MSC injections, Achilles tendons were harvested and western blot analyses were performed. Tendon tissues were frozen in liquid nitrogen and ground to a powder with mortar and pestle. They were digested in a homogeneous buffer containing 8 M urea, 50 mM Tris–HCl at pH 8.0, 1 mM dithiothreitol, and 1 mM EDTA. Extracted proteins (20 mg) were separated on 8–15% sodium dodecyl sulfate-polyacrylamide gels, and transferred to polyvinylidene fluoride membranes (Bio-Rad, Hercules, CA, USA), and blocked via incubation at 4 °C for 2 h. After that, the membranes were incubated at 4 °C overnight with anti-collagen I (ab34710; Abcam), anti-collagen III (ab7778; Abcam), and anti-VEGF antibodies (ab46154; Abcam). β-actin (ab8227; Abcam) was used as an internal control. After incubation with HRP-conjugated secondary antibody (1:3000 dilutions) at room temperature for 60–90 min, membranes were washed four times for 10 min each. Bands were visualized using ECL prime solution (Thermo Scientific, Rockford, IL, USA). We measured the intensity of the protein band using ImageJ software (NIH, Bethesda, MD, USA). The protein levels of each protein in MSCs or Pio-MSC injected tendon were normalized to those of contra-lateral control tendon. All data are from three replicates. All values are expressed as a mean ± standard error. Student's t-test was performed to compare the differences between two groups.

Supplementary Reference

S1. Lee, S. Y.; Kwon, B.; Lee, K.; Son, Y. H.; Chung, S. G., Therapeutic Mechanisms of Human Adipose-Derived Mesenchymal Stem Cells in a Rat Tendon Injury Model. *The American journal of sports medicine* **2017,** 45, (6), 1429-1439.

Supplementary Results: Immunoblotting and Animal Experiments

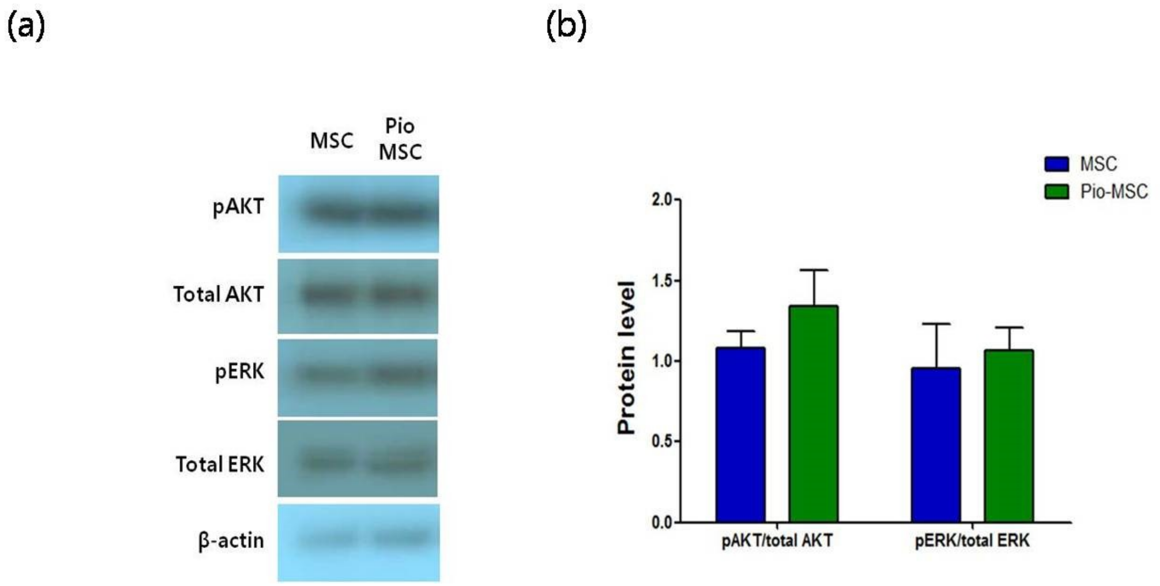

Figure S1. Immunoblotting for the detection of phosphorylated AKT andERK1/2 in MSCs and Pio-MSCs. (**a**) Non treated and treated MSCs were cultured for 1 h after pioglitazone treatment and the cell extracts were analyzed by immunoblotting for total AKT, total ERK1/2, phosphorylated AKT (Thr308), and phosphorylated ERK1/2 (Thr202/Tyr204). β-actin was used as the loading control. (**b**) Densitometric analysis of the relative level of phosphorylated AKT and ERK1/2 (Thr202/Tyr204) against their total levels in MSCs and Pio-MSCs. The values were normalized to those in the non-treated MSCs.

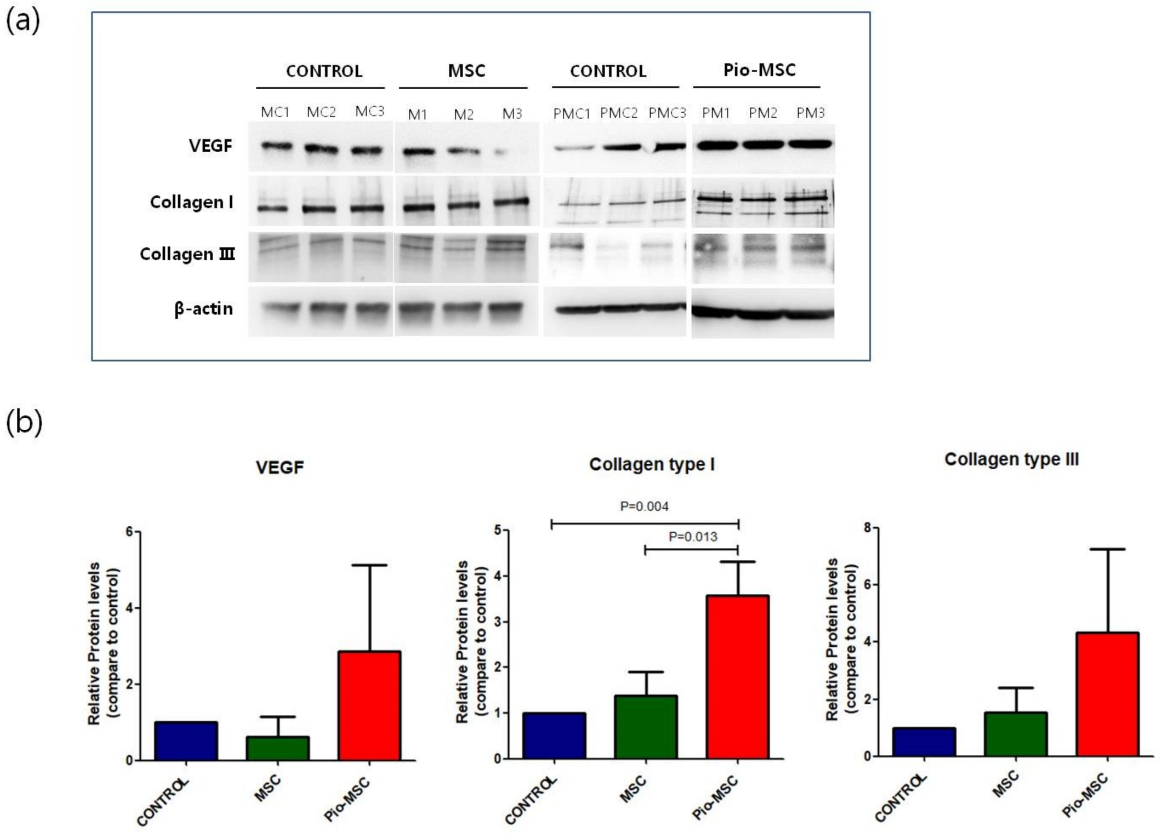

Figure S2. Immunoblotting for the detection of VEGF, collagen I, and collagen III in the rat tendon injected with MSCs or Pio-MSCs. (**a**) Tendons injected with control (PBS and fibrin glue), MSC, or Pio-MSC were isolated and the protein extracts were analyzed by immunoblotting for total VEGF, collagen I, and collagen

III. β-actin was used as the loading control. Injections were conducted two weeks prior to the analyses. (**b**) Densitometric analysis of the relative level of VEGF, collagen I, and collagen III in MSC and Pio-MSC against control animals (*n* = 3 per group).
